# Urban–Rural Disparities in the Association Between Dietary Patterns and Physical Fitness Among Chinese Children, Adolescents, and Young Adults

**DOI:** 10.3390/nu17172755

**Published:** 2025-08-26

**Authors:** Liangsen Wang, Wenyue Ma, Xinglu Li, Wenfei Zhu, Xinxin Zhang, Yuliang Sun

**Affiliations:** School of Physical Education, Shaanxi Normal University, Xi’an 710119, China; wlsen13@snnu.edu.cn (L.W.); mawnyue@snnu.edu.cn (W.M.); lixinglu@snnu.edu.cn (X.L.); wzhu@snnu.edu.cn (W.Z.); zhangxx9606@snnu.edu.cn (X.Z.)

**Keywords:** dietary patterns, physical fitness, urban–rural, children and adolescents, young adults, China

## Abstract

**Background:** This study aimed to examine the associations between dietary patterns and physical fitness among Chinese children, adolescents, and young adults, and to investigate the differences in dietary patterns and physical fitness between urban and rural Chinese children and adolescents. **Methods:** The data were obtained from the Chinese National Survey on Students’ Constitution and Health (CNSSCH). The final analysis included 200,276 participants from fourth grade to the fourth year of undergraduate study from urban and rural China. Statistical analyses were conducted using the R software (version 4.4.3). Group differences were examined using *t*-tests, and multivariable linear regression models were applied to evaluate associations between dietary patterns and physical fitness indicators, adjusting for sex, educational stage, socioeconomic status, and physical activity. Interaction terms were included to assess urban–rural differences. **Results:** Regular consumption of breakfast, eggs, and dairy products was positively associated with muscular strength, endurance, flexibility, and speed (*p* < 0.05), while higher intake of sugar-sweetened beverages was negatively associated with flexibility and muscular performance (*p* < 0.001). These associations were stronger among rural students, who benefited more from healthy dietary patterns but were also more vulnerable to the adverse effects of sugary beverages on BMI, lung capacity, and strength. **Conclusions:** This study explored the associations between dietary patterns and physical fitness among children, adolescents, and young adults in Chinese urban and rural areas. Our findings suggest that regular consumption of breakfast, eggs, and dairy products is positively associated with muscular strength, speed, endurance, and coordination, whereas frequent intake of sugary beverages is associated with poorer physical fitness. These associations appeared to differ between urban and rural populations: rural students may benefit more from nutrient-rich dietary patterns but also seem more vulnerable to the adverse associations of sugary beverages.

## 1. Introduction

Physical fitness, defined as the body’s capacity to perform physical activities effectively [1], has emerged as a critical determinant of health in children and adolescents in contemporary public health research [2]. Accumulating evidence indicates that physical fitness levels during childhood and adolescence positively correlate with immediate and long-term health outcomes, including reduced risks of obesity, improved skeletal health, and enhanced cardiovascular function [1,3]. The multidimensional construct of physical fitness encompasses cardiorespiratory endurance, muscular strength, speed, flexibility, and body composition [4], which are routinely assessed in China’s national school fitness evaluations [5,6]. This study found that dietary patterns significantly influence the development of physical fitness. Dietary pattern is a modifiable factor affecting health outcomes [7]. China’s dietary landscape has significantly transformed in recent decades, transitioning from traditional staple-based diets to more diversified patterns, such as increased high-quality protein consumption and a more nutritious breakfast [8,9]. The 2022 edition of the Chinese Dietary Guidelines similarly emphasizes the importance of regular breakfast consumption and adequate intake of high-quality protein sources (such as eggs and dairy) for school-age children, adolescents, and young adults, while discouraging excessive consumption of sugar-sweetened beverages [10,11,12]. However, across China’s diverse regions, significant dietary intake and nutritional composition variations have emerged between urban and rural children and adolescents, mainly attributable to regional development gaps, socioeconomic disparities, and differences in food availability [13,14]. Characteristically, rural residents demonstrate more traditional dietary practices, with substantially higher consumption of plant-based foods and relatively lower intake of animal-derived products than urban dwellers [8]. Importantly, these regional nutritional differences profoundly influence the health status of respective population groups [15,16]. Consequently, specialized investigations into urban–rural dietary characteristics carry substantial public health significance [17,18].

A few studies have established meaningful connections between children, adolescents, and young adults’ dietary habits and physical fitness [19,20,21,22]. Evidence indicates that consistent breakfast, eggs, and dairy consumption correlates with enhanced muscular performance and endurance capacity [23]. Conversely, excessive intake of sugar-sweetened beverages shows negative associations with cardiorespiratory function and metabolic health indicators [24,25]. One regional study conducted in Shaanxi Province further substantiates that regular intake of these nutrient-dense foods contributes to improved strength, endurance, and composite fitness scores in youth populations, with dairy products exhibiting particularly pronounced benefits [26]. However, existing studies have predominantly focused on assessing single dimensions of physical fitness in urbanized and economically developed regions, while neglecting less-developed or rural areas [18,25]. Furthermore, there is a notable absence of large-scale, nationally representative studies investigating the relationship between dietary patterns and physical fitness among urban and rural children and adolescents in China.

Therefore, this study examined the associations between breakfast frequency, egg/dairy consumption, and sugar-sweetened beverage intake with physical fitness parameters in a nationally representative sample, with comparative analyses across urban–rural populations and school stages. We proposed two main hypotheses: First, regular breakfast, eggs, and dairy products would be positively associated with better physical fitness outcomes, and frequent sugar-sweetened beverages would be negatively related to physical fitness indicators. Second, we expected that the positive effects of healthy dietary patterns (regular breakfast and egg and dairy consumption) on strength, endurance, and flexibility would be more pronounced in rural students. In contrast, the negative effects of unhealthy dietary patterns (sugared beverage consumption) might be stronger in rural compared with urban students.

## 2. Materials and Methods

### 2.1. Participants

**Figure 1:**
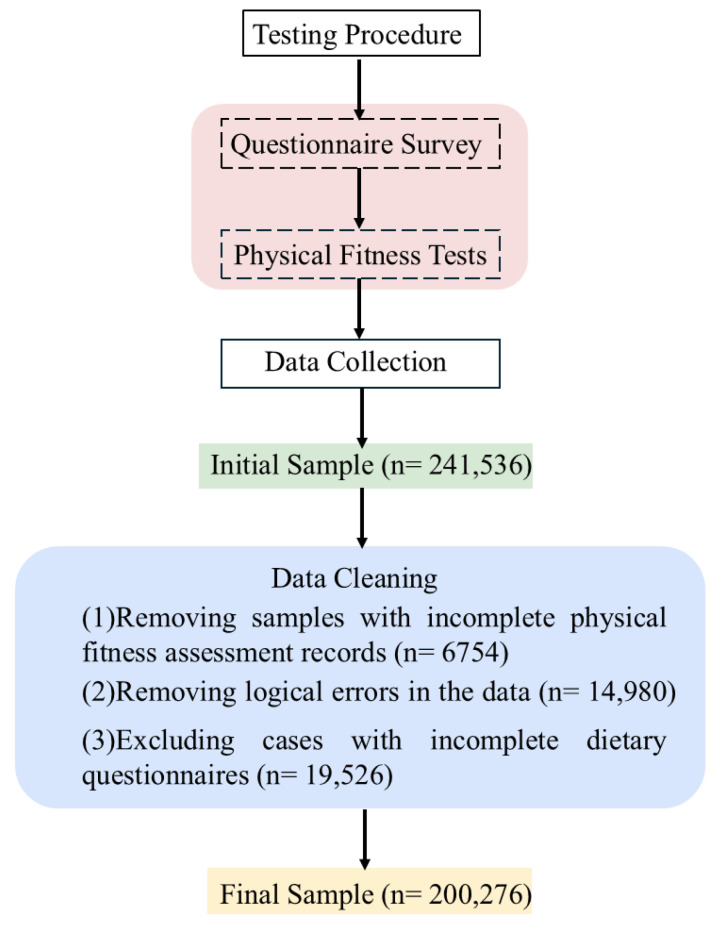
Flowchart of study design and participants’ selection process.

This study utilized data from the Chinese National Survey on Students’ Constitution and Health (CNSSCH), representing the most comprehensive nationally representative dataset on health indicators for Chinese children, adolescents, and young adults from fourth grade to the fourth year of undergraduate study [27,28,29]. In 2019, a nationally representative sample of 241,536 school children, adolescents, and young adults was selected using a multi-stage stratified sampling method. To ensure socioeconomic diversity, the sampling began by selecting the two highest-ranked and two lowest-ranked cities in terms of GDP within each province. Within these cities, counties were further chosen according to their economic level. Subsequently, schools were randomly sampled from these counties, and finally, students were randomly recruited from the official school registries. Dietary patterns and physical fitness data were collected from all participating students. Data collection occurred during the fall semester (September 2019–February 2020). Rigorous quality control procedures were implemented to exclude incomplete responses and missing data, specifically, (1) removing samples with incomplete physical fitness assessment records, (2) removing logical errors in the data, and (3) excluding cases with incomplete dietary questionnaires (Figure 1). Ultimately, 200,276 participants were included in this study. For further analysis, participants were categorized by household registration location (rural/urban), educational stage (primary school, middle school, or college), and sex (boy/girl). Primary school participants included students from grades 4 to 6 (approximately 10–12 years old); middle school participants included students from grades 7 to 12 (approximately 13–18 years old); and college participants included students from years 1 to 4 (approximately 19–22 years old). Beyond stratification by household registration (urban/rural), participants were further stratified by sex and educational stage due to differences in the mandated physical fitness test items and standards across these groups. This study was approved by the Ethics Committee of Shaanxi Normal University (201916001, 20 September 2019).

### 2.2. Data and Measurement

#### 2.2.1. Physical Fitness Assessment

Basic measurements (Table 1) included body mass index (BMI (kg/m^2^)) and forced vital capacity (mL). BMI was used as an indicator of body composition in this large-scale population study, while forced vital capacity reflects respiratory function, defined as the maximal volume of air that can be forcefully exhaled following full inspiration [30]. The physical fitness assessment comprised multiple standardized tests to evaluate participants’ performance across various domains [31]. Flexibility was assessed using the sit-and-reach test, measured in centimeters (cm). Upper body strength was assessed using the chin-up test, whereas core endurance was evaluated using the 1 min sit-up test. The standing long jump (cm) test determined lower limb explosive power. Speed was determined using the 50 m sprint test, while endurance was determined using the running test, including 1000 m running, 800 m running, and a 50 m × 8 shuttle run. Coordination was assessed using the 1 min rope-skipping test. The detailed standardized protocols, including the number of attempts and validity criteria for measurements, are presented in Table 2.

#### 2.2.2. Dietary Assessment

The dietary assessment questionnaire was derived from the standardized instruments used in China’s 2014 National Survey on Students’ Physical Health and Fitness [28]. The questionnaire was designed to accommodate the practical research conditions across diverse regions (including economically underdeveloped remote areas). Employing simplified question items ensured nationwide applicability and operational feasibility while effectively preventing low response rates or data bias that may arise from overly complex questionnaires. The survey instruments were uniformly distributed and collected, with all respondents completing them independently. For primary school participants (from the fourth to sixth grades), trained teachers provided guided assistance during the questionnaire completion. This dietary assessment module contained four key questions examining nutritional intake patterns [32,33,34,35,36]:

(1) In the past 7 days, how many days did you eat breakfast?
(2) In the past 7 days, how many days did you eat at least one egg?
(3) In the past 7 days, how many days did you drink at least one glass of milk/yoghurt or soy milk?
(4) In the past 30 days, how many times per day did you usually drink sugared beverages, such as cola, tea drinks, drinks with fruit juice, etc.?

For questions 1–3, participants selected the dietary frequency option that best matched their intake patterns, with response choices ranging from 0 to 7 days. Question 4 provided seven response categories, including “none,” “less than once daily,” “once per day,” and incremental increases up to “five or more times per day [28].

Additionally, the participants’ physical activity levels were assessed using a questionnaire consistent with the one employed in our prior research [37]. Moderate-to-vigorous physical activity (MVPA) was calculated based on the participants’ completed physical activity questionnaires. The physical activity-related section of the survey included the following questions: In the last seven days, how many of these three PA activities (light, moderate, or vigorous) did you do? What is the average number of minutes per day for each?

Gross domestic product (GDP) per capita is an essential indicator of economic development [38]. Because the questionnaire did not include household economic status information, the selected cities’ GDP per capita was used as a proxy indicator of the participants’ economic level.

### 2.3. Statistical Analysis

All statistical analyses were performed using the R software (version 4.4.3; R Foundation for Statistical Computing, Vienna, Austria). Continuous variables were first assessed for normality using kernel density estimation, and extreme outliers (z-scores > |3.0|) were excluded. Participants were grouped according to household registration (urban/rural), educational stage (primary/middle school/college), and sex (boy/girl). Independent-sample *t*-tests were applied to examine urban–rural and sex differences in dietary patterns and physical fitness indicators across these subgroups. Multivariable linear regression models were then used to analyze the associations between dietary patterns and each physical fitness indicator. The regression results were standardized regression coefficients (β), 95% confidence intervals (CIs), and *p*-values. The models were adjusted for potential confounders, including sex, educational stage, socioeconomic status, and physical activity. To further assess urban–rural disparities, interaction terms between area (urban vs. rural) and dietary patterns were included in the models, and the corresponding β, 95% CIs, and *p*-values (*p* for interaction) were reported. For fitness tests conducted exclusively among boys or girls, sex was not included as a covariate in the model.

## 3. Results

### 3.1. Participant Characteristics

This study included a total of 200,276 children, adolescents, and young adults (Table 3): at the college level, urban boys (n = 11,546 (5.8%)), rural boys (n = 11,692 (5.8%)), urban girls (n = 12,354 (6.2%)), and rural girls (n = 12,998 (6.5%)); at the middle school level, urban boys (n = 26,623 (13.3%)), rural boys (n = 26,266 (13.2%)), urban girls (n = 27,539 (13.8%)), and rural girls (n = 25,145 (12.5%)); and at the primary school level, urban boys (n = 9783 (4.8%)), rural boys (n = 11,620 (5.8%)), urban girls (n = 11,803 (5.8%)), and rural girls (n = 12,907 (6.5%)).

### 3.2. Regional and Sex Differences in Dietary Patterns and Physical Fitness

**Figure 2:**
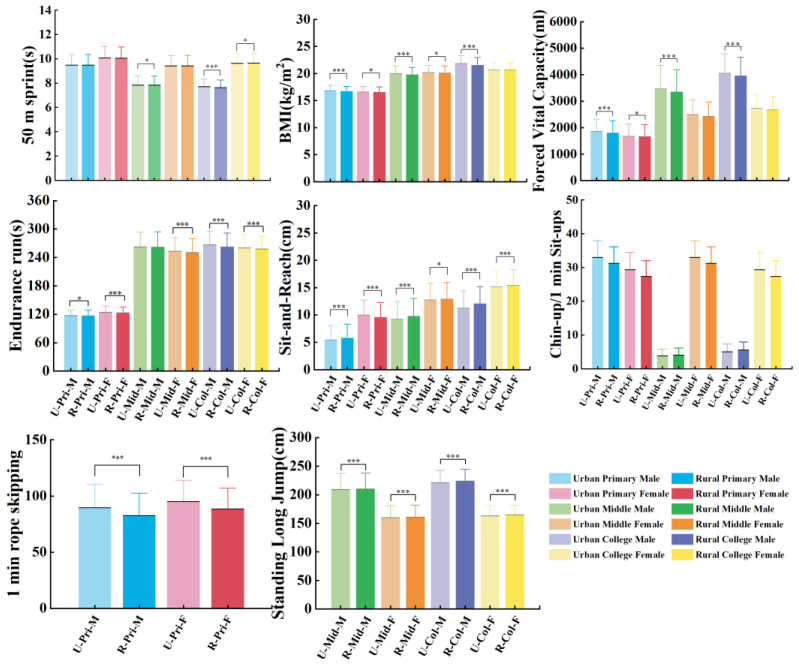
Comparison of BMI, forced vital capacity, 1 min sit-ups, chin-ups, standing long jump, endurance run, sit and reach, 50 m sprint, and 1 min rope skipping between urban and rural groups. * Indicates a significant difference (*p* < 0.05); *** indicates a highly significant difference (*p* < 0.001).

**Figure 3:**
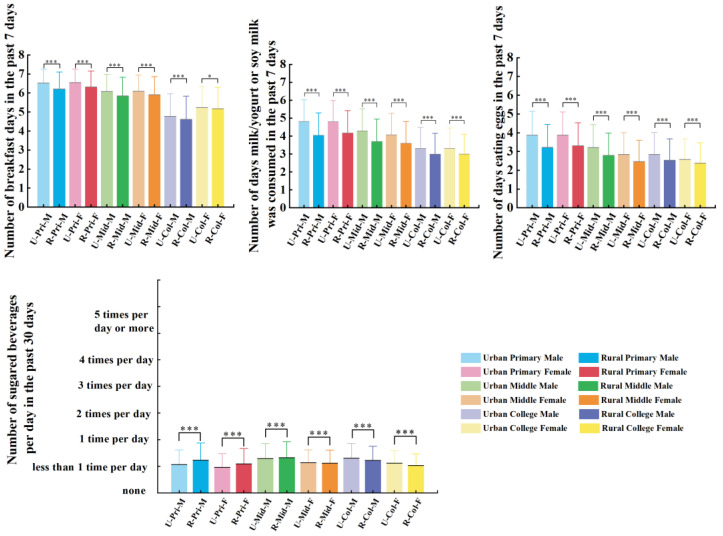
Comparison of dietary patterns between urban and rural groups. * Indicates a significant difference (*p* < 0.05); *** indicates a highly significant difference (*p* < 0.001).

There were significant differences in dietary patterns and physical fitness between urban and rural areas. All urban students reported significantly higher frequencies of breakfast, egg, and dairy product consumption than rural students (*p* < 0.001). All urban college students reported significantly higher frequencies of sugared beverages, but primary and middle urban students had lower frequencies than rural students (*p* < 0.001). Except for college girls, other urban students also reported significantly higher BMIs than rural students (*p* < 0.001). All urban students, except for middle school and college girls, reported substantially higher forced vital capacity than rural students (*p* < 0.001). Urban boys performed significantly fewer chin-ups than rural boys (*p* < 0.001). However, all urban girls performed significantly more 1 min sit-ups than rural girls (*p* < 0.001). Urban students achieved significantly shorter standing long jump distances than rural students (*p* < 0.001). Significant differences were found in endurance running performance, with rural students demonstrating faster times than urban students, except for middle school boys (*p* < 0.001). Significant differences were found in sit-and-reach performance, with rural students demonstrating greater flexibility than urban students, except for primary school girls (*p* < 0.001). We only found substantial differences in middle and college boys for the 50 m sprint; the urban boys ran faster than the rural boys (*p* = 0.039). All urban students achieved significantly better 1 min rope skipping than rural students (*p* < 0.001) (Figure 2 and Figure 3).

### 3.3. Associations Between Dietary Patterns and Physical Fitness Indicators in Urban and Rural Students

Multivariable regression analyses revealed significant associations between dietary patterns and physical fitness indicators (Table 4). Regular breakfast consumption was associated with higher BMI and better performance in the sit and reach, standing long jump, 50 m sprint, 50 m × 8 shuttle run, 800 m running, and 1000 m running. Egg consumption was positively associated with higher BMI, forced vital capacity, sit and reach, 1 min sit-ups, chin-ups, standing long jump, 50 m sprint, and 50 m × 8 shuttle run. Intake of milk, yoghurt, or soy milk was related to better performance in 1 min sit-ups, standing long jump, forced vital capacity, sit and reach, and 50 m × 8 shuttle run. Sugared beverage consumption was negatively associated with sit and reach, 1 min sit-ups, and 1 min rope skipping. Overall, healthy dietary patterns were beneficial for adolescents’ strength, endurance, and flexibility, whereas high intake of sugared beverages may adversely affect some physical fitness indicators.

The interaction analyses indicated that the associations between dietary patterns and physical fitness varied between urban and rural areas (Table 5). A significant interaction between breakfast frequency and area was observed for sit and reach, 1 min sit-ups, and forced vital capacity, indicating that the positive effects of breakfast frequency on these indicators were more potent in rural students than in urban students. A significant interaction between egg intake frequency and area was found for the 50 m × 8 shuttle run, 1000 m running, 50 m sprint, and 1 min rope skipping, indicating that the positive effects of egg intake frequency on these indicators were stronger in rural students compared with urban students. For milk, yoghurt, or soy milk intake frequency, small but significant interactions with area were observed for BMI, forced vital capacity, sit and reach, 50 m × 8 shuttle run, and 1 min rope skipping, indicating some urban–rural differences in the benefits of dairy consumption. Significant interactions were also observed between sugared beverage consumption and area for BMI, forced vital capacity, chin-ups, and standing long jump, suggesting that higher consumption of sugared beverages may have more detrimental effects on rural students than urban students.

## 4. Discussion

This study demonstrated associations between dietary patterns and physical fitness in Chinese children, adolescents, and young adults. The present study supports our initial hypotheses regarding the associations between dietary habits and physical fitness in youth. Regular breakfast consumption, egg intake, and milk, yoghurt, or soy milk intake were positively associated with multiple components of physical fitness, including strength, endurance, and flexibility. In contrast, sugared beverage consumption negatively affected several physical performance indicators. Importantly, interaction analyses revealed that these associations varied by residential area, with rural students benefiting more from healthy dietary patterns such as regular breakfast and egg consumption and being more adversely affected by higher sugared beverage intake compared with urban students.

Our findings indicate that urban students reported higher frequencies of breakfast, egg, and dairy product intake; this finding is similar to that urban students had a higher prevalence of protein intake than rural students [39,40]. The transition to a balanced diet among Chinese residents was more evident in rural areas than in urban areas during 2015–2021. However, food consumption patterns continue to exhibit significant urban–rural disparities, with urbanization, disposable income, and production levels serving as key determinants [41]. Previous studies have demonstrated that rural residents predominantly consume grain-based diets, while urban dwellers exhibit higher dairy consumption tendencies [42]. The influences on dietary pattern choices extend beyond income and price, encompassing deeply entrenched eating habits and local culinary traditions, particularly in rural areas across China [43,44]. Surveying food consumption and sources in remote areas significantly balances the dietary gap between different regions [18].

These findings partially support our hypothesis. Dietary patterns play a crucial role in the physical fitness of students [45]. Our study found that a higher intake frequency of breakfast, eggs, and dairy products was associated with better strength, speed, and endurance in students, regardless of urban–rural disparities. Breakfast was considered the most essential energy source and nutrition [46]. Our study found that children, adolescents, and young adults who ate breakfast more often tended to have better strength, speed, and endurance. This was consistent with two studies that showed that students who ate breakfast periodically had better physical fitness [47,48]. Milk and egg consumption provide additional protein and essential micronutrients, such as vitamin D [49]. Higher protein intake supplies more amino acids for muscle synthesis and may increase energy expenditure, contributing to enhanced muscular strength during exercise [50]. Dairy products also deliver critical nutrients for growing bodies, including calcium for bone health and vitamins D and A, which are necessary for overall physical development and fitness [51]. Research shows that consuming enough protein helps children and adolescents build muscle and strengthen [52], enhancing arm circumference [53]. Additionally, multiple studies have demonstrated that vitamin D improves calcium absorption, thereby supporting bone growth, maintaining bone density, and preserving muscle strength [54,55]. Our results were consistent with previous findings that higher intake of eggs and dairy products was associated with better muscle strength, endurance, and coordination. We also found that the breakfast frequency showed a positive correlation with BMI. This study emphasizes the relevance of breakfast, which provides nutrients and energy, and its omission can impair weight control. It is a factor of protection against child obesity [56,57]. That means a higher frequency of breakfast can help fight obesity. Another investigation revealed that regular breakfast intake was linked to reduced BMI and a decreased risk of overweight and obesity in both males and females [58]. Sugar-sweetened drinks—such as soda, flavored fruit beverages, energy and sports drinks, sweetened teas, and coffee—represent the primary dietary source of added sugars for children and have been linked to increased weight gain and obesity risk [59]. Almost two-thirds of children in the United States (USA) consume at least one SSB daily [60]. The overconsumption of sugary, carbohydrate-rich beverages among children and adolescents contributes to excessive calorie intake while providing minimal essential nutrients [28]. Moreover, excessive consumption disrupts the body’s natural equilibrium, compromising physiological composition and basic health needs [61]. Multiple studies have demonstrated that, beyond excessive calorie consumption, the elevated added sugar content in sweetened drinks can induce high glycemic load and exaggerated insulin response, consequently elevating obesity risk [59,62]. Our analysis revealed an inverse correlation between sugary beverage consumption frequency and key physical fitness parameters, including endurance capacity, muscular strength, and motor coordination.

We also found differences in dietary intake and physical fitness associations among urban and rural students. One study found that the adverse effects of sugary drink consumption on physical fitness differed between urban and rural students [63]. We found that the relationship between sugary drink consumption and impaired physical function appeared particularly strong among rural students, possibly due to the limited availability of healthier alternatives and insufficient awareness of healthy eating in rural areas, thereby amplifying the negative impact of high sugary beverage intake. At the same time, our findings indicated that the beneficial effects of healthy dietary patterns, such as regular breakfast and egg consumption, were more pronounced among rural students than their urban counterparts. This may be due to differences in nutritional structure and physical fitness baseline between urban and rural adolescents. In rural areas, dietary patterns are relatively monotonous, making breakfast, eggs, and dairy products more critical sources of essential nutrients and, thus, producing stronger effects [64]. Moreover, rural adolescents often engage in higher levels of physical activity than their urban counterparts, but their poorer dietary quality may make them more sensitive to nutritional improvements [65].

This study expanded the sample size based on previous research [28]. This study has several limitations that should be considered. First, these findings indicate associations between dietary patterns and physical fitness, but causal relationships cannot be inferred due to the cross-sectional study design. It provides important baseline data for future longitudinal research. Second, the simplified dietary assessment used in this study may underestimate the complexity of dietary patterns; however, this approach was chosen to ensure feasibility across diverse regions in China. Future studies should employ more comprehensive dietary assessment tools to capture nuanced nutritional behaviors. In addition, adolescent adolescence is not discussed in studies, which plays an important role in changes in physical performance.

## 5. Conclusions

This study explored the associations between dietary patterns and physical fitness among children, adolescents, and young adults in Chinese urban and rural areas. Our findings suggest that regular consumption of breakfast, eggs, and dairy products is positively associated with muscular strength, speed, endurance, and coordination, whereas frequent intake of sugary beverages is associated with poorer physical fitness. These associations appeared to differ between urban and rural populations: rural students may benefit more from nutrient-rich dietary patterns but also seem more vulnerable to the adverse associations of sugary beverages. Overall, the findings highlight the potential importance of promoting healthy eating habits in students and point to the need for nutrition interventions that consider urban–rural disparities, with particular emphasis on increasing breakfast and protein-rich food intake in rural areas. Nonetheless, given the cross-sectional nature of this study, causal inferences cannot be drawn, and future longitudinal studies are warranted to confirm these associations.

## Figures and Tables

**Table 1:** Test projects and test groups.

Test	Primary School		Middle School	College
	Grade 4	Grades 5–6		
BMI (kg/m^2^)	√	√	√	√
Forced vital capacity (mL)	√	√	√	√
1 min sit-ups (girls)	√	√	√	√
1 min sit-ups (boys)	√	√	-	-
Chin-ups (boys)	-	-	√	√
Standing long jump (cm)	-	-	√	√
50 m × 8 shuttle run (s)	-	√	-	-
800 m running (girls) (s)	-	-	√	√
1000 m running (boys) (s)	-	-	√	√
Sit and reach (cm)	√	√	√	√
50 m sprint (s)	√	√	√	√
1 min rope skipping	√	√	-	-

√ indicates required testing for this educational stage, while - indicates it is not.

**Table 2:** Methods and criteria of physical fitness test.

Component	Test Indicator	Description
Body composition	Height (cm) and weight (kg)	For body composition assessment via height and weight measurement, participants stood barefoot on a calibrated scale with heavy clothing/items removed, their backs against a vertical stadiometer (with heels, sacrum, and scapulae touching the scale), and their heads aligned horizontally, while height (to 0.1 cm) and weight (to 0.1 kg) were recorded.
Cardiorespiratory function	Forced vital capacity (mL)	The subjects should have kept standing, trying their best to inhale, aimed their mouth at the equipment’s blowing nozzle, and exhaled until they could not. Two trials were performed, with the best score retained for analysis.
Speed	50 m sprint (s)	Using a standardized standing start position, a valid 50 m dash was completed in at least two groups.
Flexibility	Sit and reach (cm)	A valid sit-and-reach test required sitting with legs fully extended, placing one hand over the other with palms facing downward, and slowly reaching forward as far as possible along the measuring scale while maintaining straight knees, measured in centimeters (cm). Two trials were performed, with the best score retained for analysis.
Lower limb strength	Standing long jump (cm)	For the standing long jump, subjects jumped with feet shoulder-width apart, with the distance measured from the take-off line to the nearest landing point. Two trials were performed, with the best score retained for analysis.
Upper body strength	Chin-ups/boys	A valid chin-up required an overhand grip on the bar, raising the chin above the bar, and returning to full arm extension. Maximal repetitions to failure with strict form were recorded.
Core strength	1-min sit-up/girls	The sit-up test counted repetitions in one minute (with hands behind the head and abdominal curl-up), maintaining 90° knee flexion throughout. Maximal repetitions completed within one minute while maintaining strict form criteria were recorded.
Endurance	50 m × 8 shuttle run(s)/1000 m/800 m run (s)	Male participants were required to complete a 1000 m run to assess endurance fitness, while female participants performed an 800 m run. Specifically, students in the 5th and 6th grades of primary school were required to complete the 50 m × 8 shuttle run. Running times were measured and recorded precisely to 1 decimal place (0.1 s).
Coordination	1 min rope-skipping test	The 1 min rope-skipping test assessed lower limb power, coordination, and endurance, with participants completing as many valid jumps as possible within 1 min on a flat surface, using an adjustable rope and a timer.

**Table 3:** Sample characteristics (n = 200,276).

		Primary School	Middle School	College	Total
Urban	Boys	9783	26,623	11,546	47,952
	Girls	11,803	27,539	12,354	51,696
Rural	Boys	11,620	26,266	11,692	49,578
	Girls	12,907	25,145	12,998	51,050

**Table 4:** Multivariable regression results of dietary patterns and physical fitness indicators.

	Diet 1			Diet 2			Diet 3			Diet 4		
Outcome	β	*p*-Value	95% CI	β	*p*-Value	95% CI	β	*p*-Value	95% CI	β	*p*-Value	95% CI
BMI (kg/m^2^)	0.01	0.015	(0.00, 0.01)	0.03	<0.001	(0.02, 0.04)	−0.01	<0.001	(−0.02, 0.00)	0.00	0.338	(−0.01, 0.00)
Forced vital capacity (mL)	−0.01	<0.001	(−0.02, −0.01)	0.05	<0.001	(0.05, 0.06)	0.01	<0.001	(0.01, 0.02)	−0.01	<0.001	(−0.01, 0.00)
Sit and reach (cm)	0.01	0.003	(0.00, 0.02)	0.01	<0.001	(0.01, 0.02)	0.01	<0.001	(0.01, 0.02)	−0.03	<0.001	(−0.03, −0.02)
1 min sit-ups	0.00	0.414	(−0.02, 0.01)	0.04	<0.001	(0.03, 0.05)	0.11	<0.001	(0.10, 0.12)	−0.02	<0.001	(−0.03, −0.01)
Chin-ups	0.01	0.123	(0.00, 0.02)	0.02	0.005	(0.00, 0.03)	0.00	0.647	(−0.01, 0.01)	0.00	0.414	(−0.01, 0.01)
Standing long jump (m)	0.01	<0.001	(0.01, 0.02)	0.01	<0.001	(0.01, 0.01)	0.02	<0.001	(0.02, 0.03)	0.00	0.079	(−0.01, 0.00)
50 m sprint (s)	−0.01	0.015	(−0.01, 0.00)	−0.01	<0.001	(−0.01, 0.00)	−0.04	<0.001	(−0.04, −0.03)	0.00	0.974	(0.00, 0.00)
50 m × 8 shuttle run (s)	−0.03	<0.001	(−0.05, −0.02)	−0.02	0.003	(−0.04, −0.01)	−0.04	<0.001	(−0.06, −0.03)	0.00	0.759	(−0.01, 0.02)
800 m running (s)	−0.05	<0.001	(−0.07, −0.04)	0.01	0.152	(0.00, 0.02)	−0.05	<0.001	(−0.06, −0.04)	0.03	<0.001	(0.02, 0.04)
1000 m running (s)	−0.05	<0.001	(−0.06, −0.04)	0.00	0.738	(−0.01, 0.01)	−0.05	<0.001	(−0.06, −0.04)	0.01	0.275	(0.00, 0.02)
1 min rope skipping	0.02	0.016	(0.00, 0.04)	0.04	<0.001	(0.03, 0.06)	0.00	0.779	(−0.02, 0.01)	−0.03	<0.001	(−0.04, −0.02)

Diet 1: In the past 7 days, how many days did you eat breakfast? Diet 2: In the past 7 days, how many days did you eat at least one egg? Diet 3: In the past 7 days, how many days did you drink at least one glass of milk/yoghurt or soy milk? Diet 4: In the past 30 days, how many times per day did you usually drink sugared beverages, such as cola, tea drinks, drinks with fruit juice, etc.? β = standardized regression coefficients.

**Table 5:** Multivariable regression analysis of interactions between dietary patterns and urban–rural area on physical fitness indicators.

	Diet 1			Diet 2			Diet 3			Diet 4		
Outcome	β	*p* for Interaction	95% CI	β	*p* for Interaction	95% CI	β	*p* for Interaction	95% CI	β	*p* for Interaction	95% CI
BMI (kg/m^2^)	0.00	0.680	(−0.01, 0.01)	0.00	0.723	(−0.01, 0.01)	0.01	0.039	(0.00, 0.02)	−0.02	<0.001	(−0.03, −0.01)
Forced vital capacity (mL)	0.01	0.001	(0.00, 0.02)	0.00	0.223	(0.00, 0.01)	0.01	<0.001	(0.01, 0.02)	−0.03	<0.001	(−0.03, −0.02)
Sit and reach (cm)	0.02	0.001	(0.01, 0.02)	−0.01	0.115	(−0.02, 0.00)	−0.01	0.029	(−0.02, 0.00)	−0.01	0.093	(−0.02, 0.00)
1 min sit-ups	0.02	0.010	(0.00, 0.03)	0.01	0.092	(0.00, 0.03)	−0.01	0.083	(−0.00, 0.00)	0.00	0.984	(−0.01, 0.01)
Chin-ups	0.01	0.458	(−0.01, 0.02)	−0.01	0.425	(−0.02, 0.01)	0.01	0.178	(−0.01, 0.03)	−0.02	0.008	(−0.03, −0.01)
Standing long jump (m)	0.00	0.518	(0.00, 0.01)	0.00	0.648	(−0.01, 0.00)	0.00	0.507	(−0.01, 0.00)	−0.01	0.015	(−0.01, 0.00)
50 m sprint (s)	0.00	0.903	(−0.01, 0.01)	0.01	0.048	(0.00, 0.01)	0.00	0.716	(−0.01, 0.01)	0.01	0.113	(0.00, 0.01)
**50 m × 8 shuttle run (s)**	0.01	0.199	(−0.01, 0.03)	0.04	<0.001	(0.02, 0.06)	0.03	0.015	(0.00, 0.05)	−0.01	0.325	(−0.03, 0.01)
**800 m running (s)**	0.00	0.560	(−0.02, 0.01)	−0.01	0.283	(−0.02, 0.01)	0.01	0.194	(−0.01, 0.03)	0.00	0.985	(−0.01, 0.01)
**1000 m running (s)**	0.02	0.029	(0.00, 0.03)	0.02	0.010	(0.01, 0.04)	0.00	0.610	(−0.01, 0.02)	0.03	<0.001	(0.02, 0.05)
**1 min rope skipping**	−0.01	0.151	(−0.04, 0.01)	0.02	0.029	(0.00, 0.04)	0.03	0.004	(0.01, 0.05)	0.00	0.981	(−0.02, 0.02)

Diet 1: In the past 7 days, how many days did you eat breakfast? Diet 2: In the past 7 days, how many days did you eat at least one egg? Diet 3: In the past 7 days, how many days did you drink at least one glass of milk/yoghurt or soy milk? Diet 4: In the past 30 days, how many times per day did you usually drink sugared beverages, such as cola, tea drinks, drinks with fruit juice, etc.? β = standardized regression coefficients. *p* for interaction refers to the *p*-value for the interaction term between dietary patterns and residential area (urban vs. rural) in the multivariable regression model.

## Data Availability

The data presented in this study are available upon request from the corresponding author. The data are not publicly available due to confidentiality reasons.
